# Cannabinoid therapeutics in orofacial pain management: a systematic review

**DOI:** 10.1111/adj.12934

**Published:** 2022-09-29

**Authors:** C Votrubec, P Tran, A Lei, Z Brunet, L Bean, BW Olsen, D Sharma

**Affiliations:** ^1^ College of Medicine & Dentistry James Cook University Smithfield Queensland Australia; ^2^ Australian Institute of Tropical Health and Medicine James Cook University Queensland Australia

**Keywords:** Orofacial pain, analgesia, cannabis, cannabinoids, cannabidiol, marijuana

## Abstract

The objective of this paper was to investigate the published evidence regarding effects of cannabinoids (natural and synthetic) on post‐operative and/or out‐of‐office pain management in patients suffering from orofacial pain that presents in the dental setting. Three online databases (Ovid (MEDLINE), PubMed (MEDLINE), Scopus) were searched (July 2021). Additional studies were sought through grey literature searching (Cochrane Library Trials and ClinicalTrials.gov) and hand‐searching the reference lists of included articles. All studies that analysed cannabinoid products and pain management of conditions that present in the general or specialist dental setting in the English language were included. Of the five articles included, one reported a significant effect on temporomandibular disorder pain relief using a topical cannabidiol formulation compared to a placebo. Four articles reported no significant effects of cannabinoids for pain management across various orofacial pain conditions. Although one study reported a positive effect, insufficient evidence exists to support a tangible clinical benefit of cannabinoids in managing orofacial pain, further research is recommended to investigate the benefits of cannabinoids’ use. © 2022 Australian Dental Association.

**Abbreviations and acronyms**
ARTG: Australian Registrar of Therapeutic Goods
BMS: burning mouth syndrome
CB1: cannabinoid receptor type 1
CB2: cannabinoid receptor type 2
CBD: cannabidiol
HNC: head and neck cancer
PRISMA: preferred reporting items for systematic reviews and meta‐analyses
QOL: quality of life
TGA: therapeutic goods administration
THC: delta 9‐tetrahydrocannabinol
TMD: temporomandibular joint disorders
TRPV1: transient receptor potential cation channel subfamily V member 1

## INTRODUCTION

Cannabis plants have been used for many millennia, as evidenced by their presence in excavated tombs dating back to the first millennium BCE.^1^, ^2^ In recent years, the focus has shifted away from the commonly known euphoric effects of cannabis towards their therapeutic potential.^3^ Specifically, cannabinoids have shown promise due to their antinociceptive, antiemetic and anticonvulsant properties.^4^ As the medicinal use of cannabinoids continue to rise throughout the world, their application for potential dental therapeutic benefit is being explored.^5^

The *Cannabis* genus of flowering plants mainly comprises the *sativa* and *indica* species.^5^ The two notable pharmacological constituents, that is cannabinoids isolated from the plant are delta‐9‐tetrahydrocannabinol (THC) and cannabidiol (CBD).^6^ THC is principally responsible for the psychoactive effects and works via activation of the endocannabinoid system. Interacting with cannabinoid type 1 (CB1) and cannabinoid type 2 (CB2) receptors, THC subsequently modulates neurotransmitter release impacting nociception, immune function, appetite and mood.^5^, ^6^, ^7^, ^8^ CBD is the non‐psychoactive component and with very low affinity for CB1 and CB2 receptors.^6^ It is thought that CBD inhibits anandamide degradation. Anandamide acts on the transient receptor potential vanilloid type 1 (TRPV1), consequently showing potent anticonvulsant, antiemetic and anti‐inflammatory properties.^8^, ^9^ These three receptors that cannabinoids activate are found across the central and peripheral nervous system in many organs and tissues, including the dental pulp and the periodontium.^5^, ^10^

Three groups of cannabinoids have been reported: ‘phytocannabinoids’ which are derived from the cannabis plant; ‘endocannabinoids’ which are naturally occurring cannabinoids synthesized in the human body that interact with cannabinoid receptors and synthetic cannabinoids.^11^ Synthetic cannabinoids agonize CB1 and CB2 receptors and typically have a greater affinity for the CB1 receptor than endocannabinoids.^11^

While cannabinoids have been legalized for medical use within the international community for some time, it was not until 29 February 2016 when changes to the Narcotic Drugs Amendment Act 2016, that the cultivation, production and distribution of medicinal marijuana were legalized in Australia. Prior to this change, Australia classed cannabinoids as a Schedule 9 Prohibited Substance.^5^, ^11^, ^12^ Currently, patients can access only two TGA‐approved medicinal cannabis products, these are CBD oil (‘Epidyolex’) for epilepsy and Nibiximols (‘Sativex’) spray on prescription from general practitioners or specialist medical practitioners according to state‐based legislation.^13^ A wide variety of unregistered cannabinoid products are also available which may also be accessed through the Therapeutic Goods Administration (TGA) under the Special Access Scheme (Category B) and the Authorised Prescriber Scheme.^13^ Not being included in the ARTG means the TGA has not assessed these products for safety, quality or effectiveness. How the dental practitioners may be able to access medicinal cannabinoids is yet to be defined at both state level and federal level.^14^ This is vital as the dentists would be the first point of contact for patients with oro‐facial pain.

In February 2021, the TGA changed the scheduling of low‐dose CBD oil (contain no more than 1% THC and used at a maximum dosage of 150 milligrams or less per day) from a Schedule 4 (S4) drug to a ‘Pharmacist Only Schedule 3 (S3) drug’.^15^ Despite this, there are currently no CBD oil products on the Australian market that fulfil the S3 requirements.^16^ Types of cannabinoid products available in Australia which are currently regulated as S4 (prescription only) and S8 drugs (controlled drugs) including floss/bud, oils, oral‐mucosal spray, liquid capsules and CBD patches, creams and gels.^12^ These products can be administered via several methods including oral‐mucosal spray, sublingual oil capsules or tablets, smoking, vaporization and trans‐dermal application.^5^, ^12^

Nabiximol (Sativex®) and cannabidiol (Epidyolex®) are the only cannabinoids currently registered on the ARTG.^17^ Sativex® is an oral‐mucosal spray with a 1:1 THC and CBD ratio from cannabis extract used to treat spasticity in multiple sclerosis patients.^13^ Epidyolex® is a plant‐derived oil‐based formulation of CBD used to treat seizures associated with refractory childhood epilepsy, such as Dravet syndrome and Lennox‐Gastaut syndrome.^13^ These medicines contain differing THC and CBD ratios.^13^ The synthetic cannabinoids dronabinol (Marinol®, Syndros®) and nabilone (Cesamet®) are licensed for medicinal use in the USA but not currently available in Australia, although have been used in Australia in the past under the SAS scheme.^5^, ^17^, ^18^

Globally, there is growing acceptance, popularity and use of cannabinoid products for a range of health conditions, including orofacial pain.^5^ Treating patients with pain in the orofacial region is one of the prominent roles that dentists play.^5^ Orofacial pain in the dental setting can include acute pain (e.g. pulpitis, apical periodontitis, post‐operative surgical pain) or chronic pain (e.g. temporomandibular disorders (TMD), burning mouth syndrome (BMS), trigeminal neuralgia).^5^ Recent studies have been carried out using *Cannabis sativa* oil to manage patients diagnosed with primary BMS, to improve the quality of life (QOL) in patients with head and neck cancer (HNC) and via topical application of CBD to treat TMD.^19^, ^20^, ^21^, ^22^ Hence, it would be very beneficial to review the current literature on the therapeutic use and effects of cannabinoids in the orofacial region.^5^ Consequently, the main objective of this systematic review was to explore the published evidence regarding effectiveness of cannabinoids in orofacial pain management in a dental setting.

## METHODS

The systematic review was carried out in accordance with the Preferred Reporting Items for Systematic reviews and Meta‐Analyses (PRISMA) statement.^23^ The clinical question ‘Are cannabinoid therapeutics effective in (acute and chronic) orofacial pain management, when compared to other pharmacological or placebo treatments’? informed the PICO framework of the systematic review, where P (population) is humans; I (intervention) is cannabinoids (natural and synthetic); C (comparison) is other pharmacological treatments or placebos and O (outcome) is improved pain management.

### Information sources and search strategy

An electronic database search using Ovid (MEDLINE), PubMed (MEDLINE) and Scopus was performed on 11 July 2021 to systematically retrieve articles. A grey literature search of ClinicalTrials.gov register and Cochrane Library Trials database was performed on the same date to search for eligible studies. No restrictions were implemented for the date or location of the publications. Databases were searched using different combinations of MeSH terms and keywords (Appendix 1). The ClinicalTrials.gov register was searched multiple times with one keyword change in each search due to user interface limitations. After record screening, citations within the included studies were also manually searched for eligible studies.

### Selection process

**Fig. 1:**
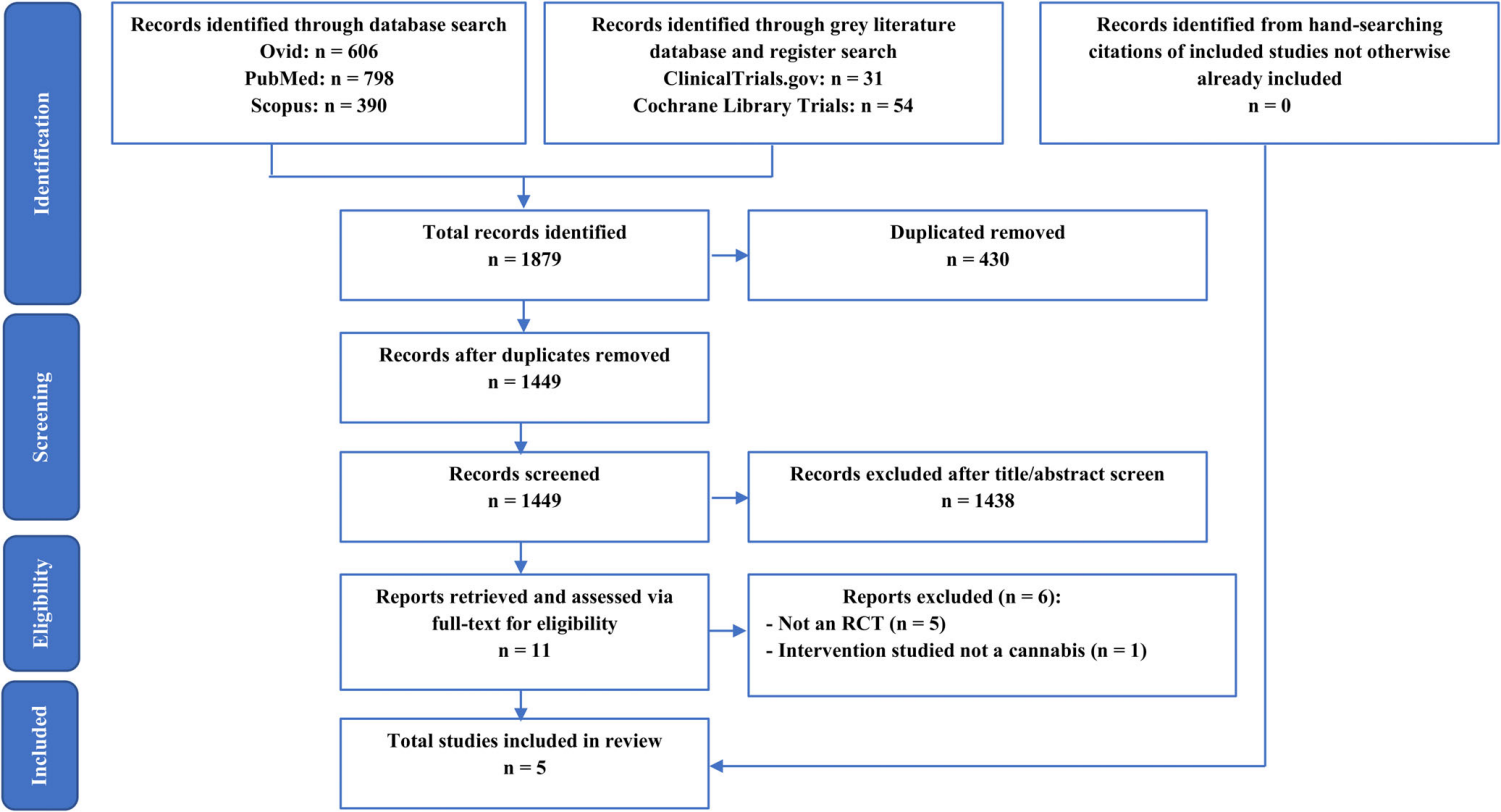
Flow chart of the systematic review according to preferred reporting items for systematic reviews and meta‐analyses guidelines.^23^

The selection process of this review was carried out as per the PRISMA flow diagram illustrated in Figure 1.^23^ EndNote^TM^ (Version 20, Clarivate Analytics) was used during the identification and screening stage to store and sort articles. Titles and abstracts of identified records were screened independently by four assessors. Conflicts in screening were discussed and resolved with mediators. Full‐text articles were then retrieved, and each report was examined by two independent assessors for eligibility for inclusion in the review. Any disagreements were arbitrated by a third independent assessor and senior researcher through discussion.

### Eligibility criteria

Studies were eligible for inclusion if they satisfied the following inclusion criteria:

- *In vivo* randomized controlled trials (RCT) involving adult humans (>18 years); published or completed with results.
- Subjects with orofacial pain (acute or chronic) as diagnosed by a dentist or dental therapist in the general or specialist dental setting.
- Investigated cannabinoids and their effect on pain management.

Studies were excluded if they were not written in the English language, involved *in vitro* or animal studies, included subjects with orofacial pain along with other conditions in which cannabinoids may influence outcomes (e.g. epilepsy, multiple sclerosis), or involved use of cannabinoid‐receptor agonists synthesized from sources other than cannabis plants. Reviews, opinion papers, case series/reports, observational studies, conference abstracts and pilot studies were also excluded.

### Data collation process

Data were extracted and compiled into a spreadsheet using a customized data form. A calibration process was used for six reviewers. Data were extracted independently from each included article by two different reviewers. Results and rationale were then reviewed by all six extractors and any disagreements were resolved by discussion and consensus was reached.

The following data items were extracted: author(s); year of publication; location of study; funding source, if identifiable; study design; sampling characteristics; measured outcome and methodology of measuring scale/device used; initial recording of measurement; follow‐up periods; adverse events and final outcomes.

### Quality and risk of bias of included studies

Articles were assessed for Risk of Bias using Version 2 of the Cochrane risk‐of‐bias tool for randomized trials (RoB 2).^24^ RoB 2 has a fixed set of bias domains, each comprising a series of signalling questions aimed to elicit information about features of trial design, conduct and reporting. Based on answers to signalling questions, a proposed judgement of ‘low’/ ‘high’ risk of bias or expressing ‘some concerns’ was generated for each domain by an algorithm.^24^ Note that the first domain is study‐level, while all other domains are outcome‐level. Additional considerations were required when assessing risk of bias in randomized crossover trials, compared to individualized‐randomized parallel‐group trials.^24^ Each article was assessed independently by two assessors and findings were collated into a spreadsheet detailing each assessor’s name, their judgement and support for judgement. The evidence level of each included study was also confirmed as per the Oxford Centre for Evidence‐Based Medicine levels of evidence.^25^ Results and rationale were then reviewed by all five remaining assessors and any disagreements were resolved by discussion and consensus reached. For studies assessed with a high risk of bias, caution was used when interpreting results in the evidence synthesis.

## RESULTS

The infographic of study selection (Figure 1) summarizes the discriminative and stepwise elimination of articles from 1879 total records down to five. The initial search of the literature was comprehensive in terminology, as well as database and grey literature navigation, yielding 1794 and 85 records respectively. After pooling of records, 430 duplicates were removed, leaving 1449 records to be screened. These records’ titles and abstracts were assessed against the inclusion and exclusion criteria (specified in methodology), which excluded 1438 studies from further appraisal as they did not meet the inclusion criteria. Full‐text articles of the remaining eleven records were retrieved and assessed for eligibility based on the inclusion criteria, from which five articles were deemed suitable for inclusion in this review. Specifically, five other reports were excluded as the study design was not an RCT, and one was excluded as the intervention was not a cannabinoid (Table 1). The study characteristics of included studies are presented in Tables 2 and 3.

**Table 1:** Summary of papers excluded and reasons for exclusion, after assessing full‐text

Author	Year	Country	Objective of the study	Reason for exclusion
Elliot *et al* ^19^	2016	USA	To better understand why patients with a history of head and neck cancer treated with radiotherapy are using medical marijuana.	Study design not an RCT.
Gambino *et al* ^20^	2021	Italy	To evaluate the use of *Cannabis sativa* oil in the management of patients diagnosed with primary burning mouth syndrome.	Study design not an RCT.
Zhang *et al* ^21^	2018	Canada	To examine the differences in ‘Quality of Life’ and psychosocial outcomes between marijuana users and nonusers with newly diagnosed head and neck cancer.	Study design not an RCT.
Ware *et al* ^35^	2002	Canada	To determine medicinal use including dose size and frequency among patients with chronic non‐cancer pain and to describe the main symptoms for which relief was sought.	Study design not an RCT.
Phan *et al* ^36^	2010	Germany	To investigate tolerance and effectiveness of cream containing cannabinoid receptor agonist, N‐palmitoylethanolamine, on burning pain from postherpetic neuralgia.	Study design not an RCT.
Marini *et al* ^37^	2012	Italy	To compare the effect of palmitoylethanolamide versus ibuprofen (NSAID) for pain relief in temporomandibular joint osteoarthritis or arthralgia.	Did not investigate a cannabis constituent or synthetic derivative of cannabis; palmitoylethanolamide is an endocannabinoid‐like lipid mediator.

**Table 2:** General characteristics of the included studies

Author	Year	Location	Objective of the study	Funding source	Study design	Measurement for pain	Initial recording	Further follow‐up post intervention	Evidence level^24^
Côté *et al* ^26^	2016	Canada	To compare the effects of nabilone versus placebo on the quality of life and side effects during radiotherapy for head and neck carcinomas	Canadian Institutes of Health Research; Fond de recherche en santé du Québec; ICN Valeant Pharmaceuticals	Randomized, double‐blind, placebo‐controlled trial	10 cm Visual Analog Scale (VAS)	Before intervention	Every 7 days during intervention and 28 days after intervention	2B
Kalliomäki *et al* ^27^	2013	USA	To evaluate the analgesic efficacy of AZD1940 in patients undergoing third molar surgical removal	AstraZeneca R&D	Randomized, double‐blind, double‐dummy, placebo‐controlled study	10 cm Visual Analog Scale	Immediately after intervention	Every 20 minutes for the first 4 hours, every 60 minutes thereafter for 8 hours	1B
Nitecka‐Buchta *et al* ^22^	2019	Poland	To evaluate the efficiency of the myorelaxant effect of CBD after the transdermal application in patients with myofascial pain	MedycynaCBD and Maciej Pawlowski for material support	Randomized, double‐blind, double‐arm, parallel‐group trial	10 cm Visual Analog Scale	Before intervention	14 days after intervention	1B
Ostenfeld *et al* ^28^	2011	UK, Italy, Germany	To evaluate the postoperative analgesic efficacy of GW824166 in patients undergoing third molar tooth extraction	No funding stated	Randomized, double‐blind, placebo‐controlled study	10 cm Visual Analog Scale, 4‐Point Categorical Verbal Rating Scale (VRS)	Before intervention and 1 hour post intervention	Every 15 minutes from 2 to 4 hours, then hourly at hour 5, 6, 7, 8, 9, 10	1B
Raft *et al* ^29^	1977	USA	To evaluate the effects of intravenous tetrahydrocannabinol in patients undergoing elective removal of four impacted third molars	National Institute of Dental Research; Division of Research Facilities and Resources	Randomized, double‐blind, placebo‐controlled crossover trial	Pain thresholds using: 1. A strain‐gauge algometer that measured periosteal pain in gram pressure (g) 2. TECA B2 EMG nerve stimulator that applied a square wave electrocutaneous stimulus to skin	Before intervention	At midpoint and 30 minutes post intervention, then at 24 hours and one month	1B

**Table 3:** Data extracted from the included articles and their major findings

Author	Sample type	Sample size and grouping	Cannabinoid type, route and frequency of administration	Measured outcome	Reported findings
Côté *et al* ^26^	Patients aged 18‐80 years with squamous cell carcinoma of the head and/or neck being treated by radiotherapy or radiochemotherapy	Total: 56 ‐ Nabilone 0.5 mg: 28 ‐ Placebo: 28 After drop‐outs ‐ Nabilone 0.5 mg: 19 ‐ Placebo: 13	Nabilone (synthetic cannabinoid) orally, 1 pill daily for first week, 2 pills daily for second week, maximum 4 pills daily from third week until end of radiotherapy	Difference in pain scores using VAS between groups	No significant difference between the 2 groups
Kalliomäki *et al* ^27^	Patients aged 18‐45 Years with a Body Mass Index (BMI) between 18‐33 kg/m^2^ and body weight between 50‐120 kg and scheduled for surgical removal of one impacted third molar	Total: 151 ‐ AZD1940 800 μg: 61 ‐ Placebo: 59 ‐ Naproxen 500 mg: 31	AZD1940 (synthetic CB1 and CB2 receptor agonist) single oral dose	Difference in pain scores using VAS between groups	No significant difference between AZD1940 and placebo
Nitecka – Buchta *et al* ^22^	Patients aged 18‐60 years in good general health with temporomandibular disorder and presence of all teeth	Total: 60 ‐ Cannabidiol 20%: 30 ‐ Placebo: 30	CBD transdermal formulation, topically twice daily for 14 days	Pain intensity changes using VAS between groups	Significant effect of CBD on average pain level and pain intensity compared to placebo
Ostenfeld *et al* ^28^	Patients aged 18‐50 in general good health and scheduled for surgical extraction of up to 4 third molar teeth	Total: 123 ‐ GW842166 100 mg pre‐operatively + placebo post‐operatively: 34 ‐ GW842166 800 mg pre‐operatively + placebo post‐operatively: 27 ‐ Ibuprofen 800 mg pre‐operatively + 400 mg post‐operatively: 31 ‐ Placebo pre‐ and post‐operatively: 31	GW842166 (selective CB2 receptor agonist) single oral dose	Pain intensity changes in VAS and VRS between groups	No significant effect of GW842166 compared to placebo
Raft *et al* ^29^	Male patients aged 18‐28 years requiring elective removal of four impacted third molars	Total: 10 ‐ THC 0.22 mg/kg: 10 ‐ THC 0.44 mg/kg: 10 ‐ Diazepam 0.157 mg/kg: 10 ‐ Ringer’s lactate (placebo): 10	THC single intravenous dose	Changes in pain detection and tolerance thresholds using strain‐gauge algometer and TECA B2 EMG nerve stimulator between interventions	No significant analgesic effect from THC compared to placebo; six subjects preferred placebo to low‐dose THC

### Risk of bias within included studies

The risk of bias within included studies was determined using the domain‐specific signalling questions in Appendix 2. Of the individually randomized, parallel‐group trials, Nitecka *et al* were judged to be of low risk of bias for all domains (Table 4).^22^ The overall risk‐of‐bias judgment for Côté *et al*’s study was high‐risk (Table 4).^26^ Studies by Kalliomäki *et al* and Ostenfeld *et al* raised some concerns, as there was risk of bias arising from the randomization process (Table 4).^27^, ^28^ The study by Raft *et al* was a crossover trial and appraised using the appropriate RoB 2 tool and noted as raising some concerns of bias (Table 5).^29^

**Table 4:** Risk of bias assessment of individual‐randomized, parallel‐group trials according to the domains of the RoB 2 Tool^24^

	Domains					
Study	1	2	3	4	5	Overall
Côté *et al* ^26^	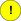	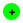	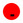	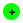	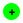	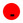
Kalliomäki *et al* ^27^	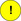	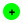	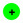	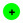	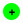	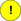
Nitecka‐Buchta *et al* ^22^	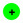	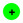	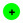	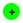	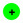	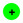
Ostenfeld *et al* ^28^	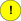	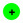	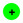	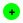	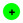	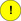

Key: 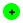 Low risk of bias, 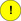 Some concerns, 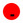 High risk of bias.

**Table 5:** Risk of bias assessment of Crossover trial according to the domains of the RoB 2 Tool^24^

	Domains						
Study	1	S	2	3	4	5	Overall
Raft *et al* ^29^	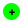	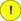	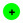	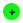	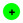	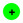	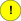

Key: 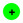 Low risk of bias, 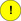 Some concerns, 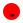 High risk of bias.

### Effects of cannabinoids on pain relief

Post‐operative orofacial pain is often a challenging form of pain to manage as it can significantly affect quality of life and delay recovery of patients. Two studies included in this review compared synthetic cannabinoids to placebos and NSAIDs (naproxen and ibuprofen respectively) with their effect on pain management following third molar extractions.^27^, ^28^ Côté *et al* also investigated a synthetic cannabinoid, nabilone, compared to placebo for HNC pain.^26^ These three studies investigated interventions that were administered orally, and none of the studies reported evidence that the synthetic cannabinoids were more effective than placebo for pain relief.^26^, ^27^, ^28^ Only study by Nitecka‐Buchta *et al* reported a statistically significant effect of CBD on pain relief compared to placebo.^22^ On the contrary, four other included studies found that the cannabinoid investigated did not produce a statistically significant pain relief when compared with placebo.^26^, ^27^, ^28^, ^29^ In fact, Kalliomäki *et al* and Ostenfeld *et al* both found NSAIDs were more effective (statistically) than placebo across all endpoints.^27^, ^28^ The remaining two studies investigated phytocannabinoids, THC and CBD.^10^ Raft *et al* investigated THC, the main psychoactive compound in cannabis, without CBD to determine if THC pain responses were due to a disruption of sensory coding or direct neural action on nociceptors.^29^ Ten subjects required removal of four impacted third molars, and each subject participated at four separate trials wherein a different premedicant was administered for each wisdom tooth over a standard 5‐minute period, after which dental surgery was commenced.^29^ Four different intravenous premedicants for third molar extractions: (1) THC (0.022 mg/kg body weight); (2) THC (0.044 mg/kg); (3) diazepam (0.157 mg/kg) and (4) placebo (Ringer’s lactate).^29^ There was no significant analgesic effect from THC compared to placebo, and six subjects preferred placebo to low‐dose THC (0.022 mg/kg).^29^ On the contrary, Nitecka‐Buchta *et al* reported statistically significant effects of CBD formulation on TMD pain relief compared to placebo, after 14 days of transdermal application over the masseter muscles.^22^ These findings may suggest that a topical route of administration of CBD is effective for pain relief as it acts on peripheral CB1/CB2 receptors with a higher local bioavailability; the bioavailability of oral CBD is limited due to digestive enzymes.^22^

All the included articles investigated patients' pain changes after administering the cannabinoid either after a specified time or over multiple time periods (Tables 2 and 3). There were variations between studies in sampling and cannabinoid used. Côté *et al* studied patients suffering from squamous cell carcinoma of the head and/or neck area and reported no significant difference in pain scores between those who received oral nabilone and placebo groups.^26^ Kalliomäki *et al* studied patients undergoing third molar extraction and divided the treatment population into three groups placebo, naproxen and an experimental synthetic CB1 and CB2 receptor agonist (AZD1940) compared to placebo.^27^ No significant differences in pain scores were noted between the two groups; however, the patients who took naproxen, a nonsteroidal anti‐inflammatory drug (NSAID), had significantly reduced pain.^27^ Ostenfeld *et al* also studied patients requiring a third molar extraction and a synthetic CB2 agonist (GW842166) at two doses (100 mg and 800 mg), compared to placebo and ibuprofen (800mg preoperative and 400mg postoperatively).^28^ They found no statistically significant difference for pain relief between placebo and 100 mg GW842166.^28^ There was improved pain relief with 800mg GW842166 but it was statistically insignificant.^28^ Notably, ibuprofen was found to be better, both statistically and clinically,at providing pain relief when compared with placebo.^28^

Raft *et al* included ten patients, each requiring removal of four third molars.^29^ THC was administered intravenously, at doses of 0.022 mg/kg and 0.044 mg/kg, with intravenous diazepam and intravenous placebo as comparison groups.^29^ Raft *et al* recorded changes in pain thresholds using a strain‐gauge algometer applied to the glabellar eminence that measured periosteal pain in gram pressure and a TECA B2 EMG nerve stimulator that applied a square wave electro‐cutaneous stimulus to skin of the acromion process.^29^ Patients responded to two psychophysical thresholds: a pain detection level and a pain tolerance level at which the stimulus intensity could no longer be withstood.^29^ Notably, no significant difference was produced between THC and placebo for pain tolerance.^29^ Furthermore, no rationale was provided for choosing intravenous route of THC administration, a route which is unfortunately not an option for the broader dental community, and thus poses issues with access and direct implication to the general dental practice.

Nitecka‐Buchta *et al* carried out a study measuring the outcome of TMJ pain after transdermal application of a CBD cream to masseter muscles.^22^ It was observed that pain intensity measured on the VAS scale after 14 days of twice daily CBD cream (Group 1) application to masseter muscles was significantly decreased from VASI = 5.6 (SD = 1.38) on Day 0, to VASII = 1.67 (SD = 1.44).^22^ The average pain level of masseter muscles after the application of the placebo formulation (Group 2) changed from VASI = 5.10 (SD = 1.26) on Day 0 to VASII = 4.60 (SD = 1.58) on Day 14.^22^ The reduction in pain intensity in VAS scale was statistically significant in Group 1 (70.2% reduction) and was not significant in Group 2 (9.81% reduction).^22^

Safety profile, tolerance and adverse events associated with cannabinoids can vary significantly between patients based on various factors, including but not limited to, the type of cannabinoid used, dosage and frequency, duration of use and comorbidities that may need additional treatment. No significant difference in adverse effects such as nausea, sleep and mood changes, drowsiness, anxiety and xerostomia were reported in the study that compared Nabilone and placebo during radiotherapy for head and neck cancers.^26^ Some of the adverse events noted in clinical trial involving AZD1940, a synthetic CB1/CB2 receptor agonist included postural dizziness, nausea, hypotension and headache and notably presyncope or syncope episodes.^27^ However, all the reported events were classed as mild to moderate in intensity and effected less than 10% of participants.^27^ Similar patterns of mild to moderate adverse events were noted in another RCT involving GW842166 wherein headache, nausea, pyrexia and syncope pharyngolaryngeal pain were reported across the study groups.^28^ In the study that utilized transdermal cannabidiol application for TMD, the authors stated that no adverse effects were recorded.^22^, ^29^ In the study using intravenous tetrahydrocannabinol, no subjects experienced true clinical psychosis, however anxiety and some dysphoria were noted on administration of THC (0.022mg/kg) in six subjects.^29^ In fact, one subject became so anxious after receiving THC (0.022 mg/kg) that surgery had to be terminated; however this subject used hashish for the previous 18 months while on active duty in Vietnam, and declared that THC recalled frightening wartime experiences.^29^ Overall, the adverse reactions occurring with these specific products trialled in the respective patient groups caused mild‐moderate adverse effects.

## DISCUSSION

As cannabinoids become more widely accepted and legally available, more research is being conducted regarding its medicinal use, particularly in pain relief and management. This can be evidenced by a range of recently published papers including a systematic review of randomized controlled trials led by the International Association for the Study of Pain (IASP) Presidential Task Force on Cannabis and Cannabinoid Analgesia.^30^, ^31^ The aforementioned paper evaluated evidence on efficacy and adverse events associated with cannabinoid use for a range of chronic pain management and concluded that a very low‐quality evidence exists supporting their efficacy and significant adverse events were reported with their usage on short term (<7 days) and longer term usage (>7 days).^30^, ^31^

In a recent anonymous online cross‐sectional survey conducted in Australia 2 years after legal access was introduced (in 2016), less than 3% of the respondents (25 out of 1044) had obtained cannabinoids through legal prescription which was attributed to lack of awareness of access through medical practitioner.^32^ This paper and an earlier report by the same group highlight the need for further research within this expanding option in pain management.^32^, ^33^ However, exponential growth in popularity of cannabinoid usage over the last 4 years, specifically in cancer patients has significantly increased awareness. Furthermore, limited scientific literature around its use in dentistry have been published possibly due to restricted access and lack of compelling evidence for its efficacy, as noted in this review.

In our review, five publications that evaluated the use of cannabinoids on pain management, with varying results, were identified and appraised. Although all the included studies in the analysis were human studies, variations in sample populations, gender differences in study population, type of cannabinoid, routes of administration, and outcome measurements contributed to the heterogeneity of included studies. This presents difficulties when attempting to draw direct comparisons between studies to formulate concise conclusions.

Effective orofacial pain management is often achieved using a combination of anti‐inflammatory, steroids and NSAIDs. Of the studies included in our review, statistically significant pain relief was noted for TMD pain upon topical application of a transdermal CBD formulation over the masseter muscles. Its benefit may be derived from its action on peripheral CB1/CB2 receptors with a higher local bioavailability by avoiding the gut.^22^ This approach presents a new avenue for chronic pain management that avoids the adverse effects of first‐pass metabolism following enteral administration, especially in cases where higher drug concentrations are required to achieve a sufficient systemic bioavailability.^22^ However, the concerns around the adequacy (or lack of) oral formulations of the synthetic cannabinoids investigated by three other included studies to impart analgesic effects still exist.^26^, ^27^, ^28^ Additionally, very low and suboptimal intravenous drug concentrations use was a potential limitation in the study involving THC.^29^

Methodology of recording outcome measures did not differ between studies, aside from Raft *et al*.^29^ Pain was measured and recorded utilizing the Visual Analog Scale by most studies.^22^, ^26^, ^27^, ^28^As such, the quality of these studies in terms of outcome measurements were relatively equivalent despite the VAS score being a relatively blunt instrument. However, Raft *et al* recorded changes in pain detection and pain tolerance thresholds after drug administration using a cutaneous and pressure stimulus, as previous discussed.^29^ Studies included in this review treated different types of pain, which contributes to the variation between studies. Furthermore, four included studies reported pain intensity as their outcome measure whilst only Raft *et al* ^29^ assessed pain tolerance. Three studies treated patients requiring third molar extractions and sought to manage the associated post‐operative pain.^27^, ^28^, ^29^ Côté *et al* included patients that were undergoing radiotherapy and/or chemotherapy for squamous cell carcinoma, while Nitecka‐Buctha *et al* studied patients that suffered from TMD pain.^22^, ^26^ These differing patient types with varying causes of pain may result in different reported pain and relief, ultimately impacting measured outcomes and the ability to draw comparisons. For instance, the acuity and chronicity of pain differed between studies, as third molar extractions typically result in acute pain compared to TMD and cancer, which may have a chronic element of pain.^22^, ^27^ Furthermore, pain can be a personal and subjective experience, mainly when anxiety, certain perceptions or past experiences are involved.^34^ Consequently, tailored research may need to be pursued for each of these types of pain and evaluate how different cannabinoids, pain perceptions and experiences affect pain relief.

The included studies had a range of limitations. The sample size in Côté *et al* study was relatively small (56 patients) and from a single centre.^26^ Twenty‐four participants dropped out, seemingly related to their health status, therefore, it is possible that the absence of outcome data depended on its true value (Domain 3, Table 4); this gives rise to loss‐to‐follow‐up bias.^24^ Additionally, there were some concerns in the randomization process as there was an unbalanced distribution of patients with advanced carcinoma, receiving combined modality treatments (radiochemotherapy) were unequally represented in both groups (Domain 1).^24^, ^26^ This could explain higher dropouts from the placebo group, or alternatively there may have been less dropouts from the nabilone group due to the benefits of this intervention—neither conclusion can be drawn with certainty. Studies by Kalliomäki *et al* and Ostenfeld *et al* raise some concerns of bias as random component used in sequence generation, nor was the concealment of allocation sequence (Domain 1, Table 4) was included.^27^, ^28^ Raft *et al*’s crossover trial raised some concerns of bias due to inadequacies of information surrounding period and carry‐over effects (Domain S, Table 5).^24^, ^29^ Additionally, despite a homogenous sample that did not differ in their pre‐surgical anxiety ‘trait’ levels, one subject had to be excluded because they became so anxious after injection of THC (0.022 mg/kg) that surgery had to be terminated.^29^ However, missing outcome data from one participant in one intervention are quite small to have any significant difference on the estimated effect of intervention.^29^ In addition, the measurement of pain threshold /tolerance as an outcome instead of pain relief made it difficult to compare this study with others.

This paper sought to review RCTs of synthetic and natural cannabinoids. The inclusion criteria were expanded to include all these cannabinoids due to the known ethical barriers associated with investigating marijuana use in subjects. Moreover, the legality of recreational or medicinal marijuana widely varies per jurisdiction.^5^, ^13^ As a result, none of the included studies investigated cannabinoid use by inhalation or sublingual administration. There are some non‐randomized studies investigating the benefits of marijuana for HNC patients, however, these subjects were mostly existing marijuana users.^19^, ^21^ Hence, this review supports the recommendation that further studies are needed to investigate the benefits of cannabinoids in different formulations to inform future policy changes surrounding its use as a dental therapeutic. While four out of five studies included in this review failed to find significantly positive results, some studies that were excluded from this review (due to non‐RCT design) reported a positive association between cannabinoid use and pain relief. In a recent prospective, open‐label, single‐arm pilot study, Gambino *et al* evaluated the use of *C. sativa* oil in the management of patients diagnosed with primary BMS.^20^ The subjects showed a statistically significant improvement in the clinical remission of oral symptoms over time.^20^ Levels of anxiety and depression also demonstrated a statistically significant and favourable improvement with no reports of severe reactions.^20^ One‐third of the patients experienced adverse events; nevertheless, no subjects had to discontinue the treatment due to these adverse events, which were all transient.^20^ The most common adverse event reported was dizziness occurring in three out of 17 patients (17.6%), followed by headache (11.8%) and constipation (5.9%).^20^ Overall, the treatment was well‐tolerated and effective in patients similar to some studies included in this review.^20^

While Côté *et al* did not find nabilone to be effective at improving the QOL of HNC patients, other non‐randomized studies concluded that marijuana use did lead to pain relief in HNC patients.^26^ Elliot *et al* carried out a cross‐sectional study investigating why patients with a history of HNC treated with radiotherapy were using medical marijuana.^19^ Using four different QOL questionnaires in their sample of 15 patients, they found that medical marijuana subjectively assisted with pain management and other long‐term side effects of radiotherapy, including altered sense, appetite, depression, dysphagia, and xerostomia.^19^ Zhang *et al,* in a prospective cohort study, compared similar QOL measures in recreational marijuana users case‐matched with nonusers who were newly diagnosed with HNC and treated in a tertiary setting.^21^ They supported Elliot *et al*'s findings, with statistically significantly lower pain scores in marijuana users (loose‐leaf marijuana) than nonusers.^21^ Similarly, statistically significant improvement was found for anxiety/depression, tiredness, drowsiness, appetite, and general well‐being measured using (Edmonton Symptom Assessment System (ESAS) score).^21^ However, Elliott *et al*’s study had a small sample size and both studies relied on samples of historical users of marijuana, which limits the external validity of the results.^19^, ^21^ These factors may contribute to the differences in the randomized, double‐blind, placebo‐controlled HNC trial performed by Côté *et al*. Conversely, it should be acknowledged that other constituents and effect(s) of cannabinoids may be responsible for reduced pain perception, as compared to the synthetic cannabinoid studied by Côté *et al* which mainly agonizes CB1/CB2 receptors.^6^, ^26^

## CONCLUSIONS

The cannabinoids CBD and THC exhibit a wide spectrum of antinociceptive and anti‐inflammatory actions with a range of side effects.^4^, ^5^, ^20^ Theoretically, there is great potential to use cannabinoids in the management and treatment of orofacial pain, however, its use in healthcare remains controversial and in its infancy.^5^ Specifically, THC as an intravenously delivered cannabinoid has limited potential for use in dental setting due to dentists rarely using intravenous mode of medication delivery and risk of psychogenic effects. Generally, a low‐quality evidence supporting the use of cannabinoids to treat pain and inflammation exist, with a lack of consistent and compelling high‐quality evidence pertaining to its effectiveness in orofacial pain. Although one study in this review reports positive effects, insufficient evidence exists to support a tangible clinical benefit of natural and synthetic cannabinoids in managing orofacial pain, especially for drugs delivered into systemic circulation.^22^ Despite the one study demonstrating topical CBD may be beneficial for treating orofacial pain, further research is needed prior to its use. Future research in the form of rigorous randomized studies, including crossover RCTs to evaluate varying doses of topical and systemic cannabinoids and possible interactions with other medications is suggested. As the therapeutic benefits of natural and synthetic cannabinoids continue to evolve, dental professionals will need to be familiar with the potential indications for use and possible interactions in the practice setting in an era of constantly changing legislation.

### REGISTRATION AND PROTOCOL

This review was carried out in accordance with the PRISMA guidelines^23^ and registered with PROSPERO 2022 Registration number CRD42022274854 available from: https://www.crd.york.ac.uk/prospero/display_record.php?ID=CRD42022274854

## DISCLOSURE

Nothing to declare.

## Appendix 1 Databases searched and corresponding MeSH terms used.

**Table:**

Electronic Database	MeSH terms and keywords search
Ovid	exp Dentistry/ or exp Stomatognathic Diseases/ or exp Pharyngeal Diseases/ or exp neck/ or exp facial bones/ or exp jaw/ or pterygopalatine fossa/ or exp sphenoid bone/ or exp temporal bone/ or exp temporomandibular joint/ or exp stomatognathic system/ or exp pharynx/ or exp facial nerve/ or glossopharyngeal nerve/ or exp trigeminal nerve/ or "head and neck neoplasms"/ or exp otorhinolaryngologic neoplasms/ or exp maxillary sinus/ or exp Odontogenic Tumors/ Cannabis/ or exp Cannabinoids/ or exp "Marijuana Use"/ or Medical Marijuana/ or Marijuana Abuse/ or exp Receptors, Cannabinoid/ or exp Endocannabinoids/ exp Pain/ or Pain Management/ or exp "Anesthesia and Analgesia"/ or exp analgesics/ or exp muscle relaxants, central/ or exp Inflammation/ or exp Anti‐Inflammatory Agents/ or exp somatosensory disorders/ or exp Pain Perception/ or Pain Measurement/ or Nociceptors/ (dent* or orofacial or "oral medicine" or "oral pathology" or "oral surgery" or temporomandibular or trigeminal or molar or tooth* or teeth or myofunctional or periodont* or endodont* or (osteonecrosis and jaw) or periapical or "radicular cyst" or "burning mouth" or "dry socket" or "alveolar osteitis" or stomatitis or "oral mucositis" or jaw or mandib* or maxilla* or masticat* or "oral mucosa" or "oral ulcers" or masseter or tongue or saliva* or pharyn* or (head and neck) or "alveolar bone" or temporalis or oropharyn* or labial* or lip or pulp or palate or palatal or odont*).mp. [mp=title, abstract, original title, name of substance word, subject heading word, floating sub‐heading word, keyword heading word, organism supplementary concept word, protocol supplementary concept word, rare disease supplementary concept word, unique identifier, synonyms] (cannab* or marijuana or hemp or nabilone or dronabinol or THC or tetrahydrocannabinol or CBD or nabiximol or sativex or nanabis).mp. [mp=title, abstract, original title, name of substance word, subject heading word, floating sub‐heading word, keyword heading word, organism supplementary concept word, protocol supplementary concept word, rare disease supplementary concept word, unique identifier, synonyms] (pain or analges* or anaesthe* or inflammat* or "muscle relaxant" or anti‐inflammatory or antiinflammatory or nocicept* or ache or antinocicept* or hyperalgesia or allodynia or neuropathic pain).mp. [mp=title, abstract, original title, name of substance word, subject heading word, floating sub‐heading word, keyword heading word, organism supplementary concept word, protocol supplementary concept word, rare disease supplementary concept word, unique identifier, synonyms] 1 or 4 2 or 5 3 or 6 7 and 8 and 9
PubMed	(cannab* or marijuana or "cbd" or "thc" or nabilone or hemp or nabiximol or sativex or dronabinol) AND (dent* or orofacial or tooth* or head or neck or odont* or teeth or jaw or stomatitis or trigeminal) AND (pain or analges* or anaesthe* or inflammat* or “muscle relaxant” or antiinflammatory or anti‐inflammatory or nocicept* or antinocicept* or hyperalgesia or allodynia or neuropathic pain)
Scopus	(dent* OR "third molar" OR "tooth extraction" OR pulp OR toothache OR "oral cancer" OR "burning mouth" OR "trigeminal neuralgia" OR "oral mucositis" OR "head and neck" OR temporomandibular OR odont* OR “dry socket” OR “alveolar osteitis” OR “oral surgery” OR stomatitis OR pharyn* OR jaw OR orofacial) AND (cannab* OR marijuana OR hemp OR dronabinol OR "illicit drug" OR cbd OR thc OR nabilone OR nabiximol OR sativex OR tetrahydrocannabinol) AND (pain OR analges* OR anaesthe* OR inflammat* OR “muscle relaxant” OR anti‐inflammatory OR antiinflammatory OR nocicept* OR "neuropathic pain" OR hyperalgesia OR allodynia)
ClinicalTrials.gov	Cannabis | Completed Studies | Studies with Results | *insert keyword consecutively from list 1‐13 below* Head and Neck Cancer Pain Burning Mouth Trigeminal Neuralgia Oral Mucositis Temporomandibular Dry Socket Alveolar Osteitis Stomatitis Orofacial Odontalgia Third Molar Dental
Cochrane Library Trials	(cannab* or marijuana or "cbd" or "thc" or nabilone or hemp or nabiximol or sativex or dronabinol) AND (dent* or orofacial or tooth* or head or neck or odont* or teeth or jaw or stomatitis or trigeminal or temporomandibular) AND (pain or analges* or anaesthe* or inflammat* or “muscle relaxant” or antiinflammatory or anti‐inflammatory or nocicept* or antinocicept* or hyperalgesia or allodynia or neuropathic pain)

## Appendix 2 Cochrane RoB 2 Tool signalling questions for assessment of individual‐randomized, parallel‐group trials and for assessment of crossover trials.^23^

**Table:**

Domain	Signalling questions for individual‐randomized, parallel‐group trials	Signalling questions for crossover trials
1. Randomization process		
1.1	Was the allocation sequence random?	
1.2	Was the allocation sequence concealed until participants were enrolled and assigned to interventions?	
1.3	Did baseline differences between intervention groups suggest a problem with the randomization process?	Did baseline differences between intervention groups at the start of the first period suggest a problem with the randomization process?
S. Bias arising from period and carry‐over effects		
S1	n/a	Was the number of participants allocated to each of the sequences equal or nearly equal?
S2	n/a	If N/PN/NI to S.1: Were period effects accounted for in the analysis?
S3	n/a	Was there sufficient time for any carryover effects to have disappeared before outcome assessment in the second period?
2. Deviations from intended interventions		
2.1	Were participants aware of their assigned intervention during the trial?	Were participants aware of their assigned intervention during each period of the trial?
2.2	Were carers and people delivering the interventions aware of participants' assigned intervention during the trial?	Were carers and people delivering the interventions aware of participants' assigned intervention during each period of the trial?
2.3	If Y/PY/NI to 2.1 or 2.2: Were there deviations from the intended intervention that arose because of the trial context?	
2.4	If Y/PY to 2.3: Were these deviations likely to have affected the outcome?	
2.5	If Y/PY/NI to 2.4: Were these deviations from intended intervention balanced between groups?	If Y/PY/NI to 2.3: Were these deviations from intended intervention balanced between interventions?
2.6	Was an appropriate analysis used to estimate the effect of assignment to intervention?	
2.7	If N/PN/NI to 2.6: Was there potential for a substantial impact (on the result) of the failure to analyse participants in the group to which they were randomized?	
3. Missing outcome data		
3.1	Were data for this outcome available for all, or nearly all, participants randomized?	
3.2	If N/PN/NI to 3.1: Is there evidence that the result was not biased by missing outcome data?	
3.3	If N/PN to 3.2: Could missingness in the outcome depend on its true value?	
3.4	If Y/PY/NI to 3.3: Is it likely that missingness in the outcome depended on its true value?	
4. Measurement of the outcome		
4.1	Was the method of measuring the outcome inappropriate?	
4.2	Could measurement or ascertainment of the outcome have differed between intervention groups?	Could measurement or ascertainment of the outcome have differed between interventions within each sequence?
4.3	If N/PN/NI to 4.1 and 4.2: Were outcome assessors aware of the intervention received by study participants?	
4.4	If Y/PY/NI to 4.3: Could assessment of the outcome have been influenced by knowledge of intervention received?	
4.5	If Y/PY/NI to 4.4: Is it likely that assessment of the outcome was influenced by knowledge of intervention received?	
5. Selection of the reported result		
5.1	Were the data that produced this result analysed in accordance with a pre‐specified analysis plan that was finalized before unblinded outcome data were available for analysis?	
5.2	Is the numerical result being assessed likely to have been selected, on the basis of the results, from multiple eligible outcome measurements (e.g. scales, definitions, time points) within the outcome domain?	
5.3	Is the numerical result being assessed likely to have been selected, on the basis of the results, from multiple eligible analyses of the data?	
5.4	n/a	Is a result based on data from both periods sought, but unavailable on the basis of carryover having been identified?
